# Liquid-liquid extraction intensification by micro-droplet rotation in a hydrocyclone

**DOI:** 10.1038/s41598-017-02732-x

**Published:** 2017-06-02

**Authors:** Yuan Huang, Hua-lin Wang, Yu-quan Chen, Yan-hong Zhang, Qiang Yang, Zhi-shan Bai, Liang Ma

**Affiliations:** 1 State-Key Laboratory of Chemical Engineering, East China University of Science and Technology, Shanghai, 200237 PR China; 2 PetroChina Karamay Petrochemical Company, Kelamayi, 834003 PR China

## Abstract

The previous literature reports that using a hydrocyclone as an extractor intensifies the mass transfer and largely reduces the consumption of extractant from 1800–2000 kg h^−1^ to 30–90 kg h^−1^. However, the intensification mechanism has not been clear. This paper presents experimental and numerical methods to study the multi-scale motion of particles in hydrocyclones. In addition to the usually considered translational behavior, the high-speed rotation of dispersed micro-spheres caused by the anisotropic swirling shear flow is determined. The rotation speeds of the tested micro-spheres are above 1000 rad s^−1^, which are much larger than the instantaneous rotation speed in isotropic turbulence. Due to the conical structure of a hydrocyclone, the rotation speed maintains stability along the axial direction. Numerical results show that the particle Reynolds number of micro-droplets in a hydrocyclone is equal to that in conventional extractors, but the particles have high rotation speeds of up to 10,000 rad s^−1^ and long mixing lengths of more than 1000 mm. Both the rotation of micro-droplets along the spiral trajectories and the intense eddy diffusion in a hydrocyclone contribute to the extraction intensification.

## Introduction

Liquid-liquid extraction is an important method for heterogeneous separation. There are dozens of extraction devices, such as mixer-settlers^1^, agitated extraction columns^2^, spray sieve plate columns^3^, rotating disc columns^4^, centrifugal extractors^5^, etc. To improve the extraction efficiency and decrease the consumption of the solvent and the cost of production, research efforts usually focus on the intensification of the mass transfer process for extraction devices. The phase ratio, the ratio of the solvent to the feed solution, of conventional extraction devices is usually above 0.1 (Table 1). The use of a large volume of solvent results in a huge consumption of energy in the subsequent distillation or reverse extraction process. In fact, since 1842 when Peligot first found that uranyl nitrate can be isolated from nitric acid solution by diethyl ether, it has been the focus and difficulty of many research efforts to reduce the phase ratio. Wang^6^ and Wu^7^ have reported using a hydrocyclone as an extraction device to isolate amine and alkali impurities from the C_4_ feed of an MTBE unit of 20,000 t a^−1^ in the PetroChina Karamay Petrochemical Company. Compared to the former mixer-settler device, the water extractant dosage was reduced from 1800–2000 kg h^−1^ to 30–90 kg h^−1^

**Table 1:** The phase ratio of familiar extraction devices reported.

Author	Extractor type	Chemical system	Phase ratio, E/M	Extraction efficiency
Zamponi *et al*.^41^	stirred extraction column	Toluene/acetone(0.045 kg kg^−1^)/water	1.28	85.7%
Lee *et al*.^42^	Agitated extraction column	Kerosene and paraffin oil/penicillin G potassium salt(0.408 mol L^−1^)/citrate buffer solution	2	≈85%
Laitinen *et al*.^2^	agitated extraction column	Supercritical carbon dioxide/1−butanol(5 wt%)/water	2.7	99.7%
Benz *et al*.^43^	Mixer-settler	Toluence/acetone/water	1	96%
Dehkordi^44^	Mixer-settler	Cumene/iso−butyric acid/water	1	99%
Abdeltawab *et al*.^45^	Stirred extraction column	2-ethylhexyl phosphonic acid mono-2-ethylhexyl ester/La and Ce nitrates/nitric acid	0.31	30% (La) 90% (Ce)
Serrano−Purroy *et al*.^46^	Centrifugal extractor	N,N’-dimethyl-N,N’-dioctyl-2-(2-(hexyloxy)ethyl)-malonamide/MOX fuel/HNO_3_	1	97.8% (Tc)
Zhu *et al*.^47^	annular centrifugal extractor	Ionic liquid/octane/ethylbenzene	0.5~2	95%(single stage)
Zhou *et al*.^48^	Annular centrifugal extractor	R_3_N and (R_3_NH)_2_SO_4_/*p*-cresol-HNO_3_/water	1/7.6~1	92.01%~99.98%
Gameiro *et al*.^49^	Pulsed sieve-plate column	Shellsol D-70/NH_3_/(NH_4_)_2_SO_4_	0.5~5	90.5%~99.5%
Yung *et al*.^50^	Reciprocating plate column	Ionic liquids/phenol/water	0.24~1.05	
Zhao *et al*.^51^	Annular centrifugal extractor	TRPO-kerosene/ Fe^3+^/ HNO_3_	0.32~3.03	60%(3-stage)
Birajdar *et al*.^52^	Bubble column	n-butanol/2,3-butanediol/water	0.8	54.0%
Modak *et al*.^53^	rotating packed bed contactor	Xylene/methyl red/water	0.04~0.10	97%~98%
Ashrafmansouri *et al*.^54^	spray extraction column	Toluene/acetic acid/water	14	75%

, corresponding to a phase ratio reduced from 0.15–0.17 to 0.0025–0.0075. Furthermore, the operating period was prolonged from 15 days to 35–45 days, and the annual consumption of catalyst decreased from 15.1 t to 6.3 t. However, the intensification mechanism of extraction by hydrocyclone has proven difficult to explain. This study attempts to understand it by investigating the surface movement of the micro-droplet (particle rotation) with a solid micro-sphere as a substitute.

The motion of suspended particles is sufficiently affected by the surrounding fluid. The most obvious sense of motion is their migration following the flow. However, in addition to this, like the leaves change their posture in the air when they fall from a tree, the suspended particles rotate as they are subjected to the forces generated by the surrounding shear flow^8^. Many research studies have been devoted to solid particle rotation in simple shear flow, studies of aspects such as the forces acting on particles^9, 10^, the radial displacement in tubes with Poiseuille flow^11–15^ and migration in Couette flow between two relative moving parts^16–19^. Because of the complex particle motion in turbulent flow, the measurement of micro-particle rotation has for a long time been limited to simple shear flow^14, 20–23^. However, some new high speed imaging systems for rod-like particles rotating in isotropic turbulent flows have been recently developed^24–27^. In addition, Klein *et al*.^28^ and Meyer *et al*.^29^ have measured the rotation of spheres (7 cm and 8 cm in diameter, respectively) in isotropic turbulent flows.

Since the translational speed (2–10 m s^−1^) of micro-particles in a hydrocyclone is much faster than that (much less than 1 m s^−1^) in the isotropic turbulent flows reported in the literature, it is difficult to track the particle’s motion behavior, especially to identify its rotation. This study used the solidified emulsion droplets that were fabricated in a microfluidic device and have double black cores as the tracer microspheres. Utilizing the high-speed imaging technique, the rotation speeds of micro-spheres were then measured.

Because it is difficult to directly measure the rotation of micro-droplets in turbulent flow, numerical simulation is usually a convenient and reliable method to predict their rotation speed. According to previous studies^30–34^, direct numerical simulation (DNS) has a good precision of predicting the rotation speed of particles in low Reynolds number turbulence. However, due to its high requirements of time and spatial resolution, it is unsuitable to use DNS to calculate the 3D turbulent flow in a hydrocyclone at the current computing levels. The Reynolds stress model (RSM) and large eddy simulation (LES) are usually used to calculate the velocity field of the complex swirling flow in hydrocyclones because their results are in good agreement with the experiments^35, 36^. In addition, according to Jeffrey’s theory, the rotation speed of particles can be calculated by ***ω*** = **Ω**/2^8, 20^, when their diameters are smaller than the smallest length scale of the eddy and when their density matches that of the fluid. The vorticity **Ω** of the field is calculated by **Ω =** ▿ × **u**. Considering the cost of simulation, this study applied RSM to calculate the velocity field in a hydrocyclone and then calculate the sphere rotation speed.

## Results

### Experimental design

**Figure 1:**
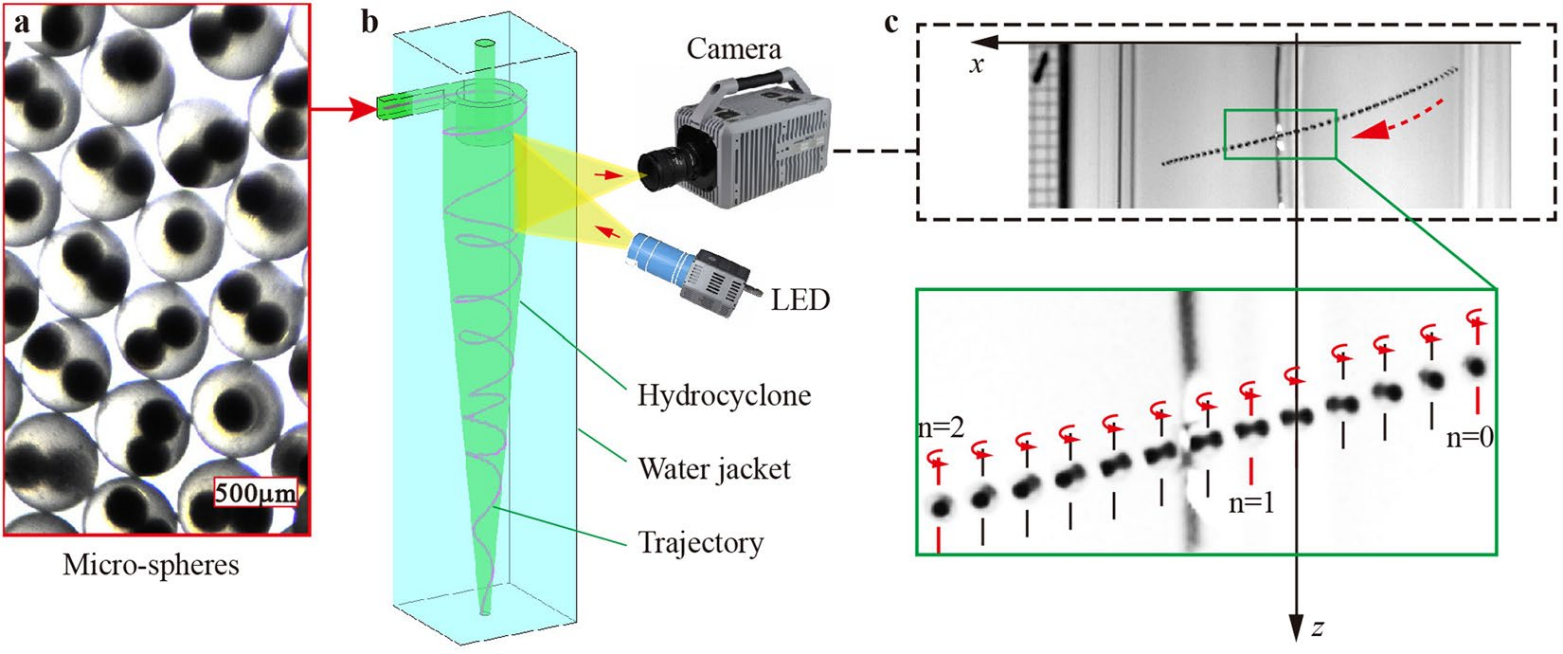
The principle of identifying testing micro-sphere rotation. (**a**) Optical image of micro-spheres that have two black cores. (**b**) Schematic diagram of the measuring process of micro-sphere rotation with a high-speed camera. (**c**) Image of a micro-sphere rotation captured by high-speed camera. The letter n represents the number of times that the micro-sphere rotates *π*/2.

The schematic of detecting micro-spheres rotating in the hydrocyclone is shown in Fig. 1. The highly monodisperse micro-spheres, which have a transparent shell (470 μm) and double black spherical cores (200 μm) (Fig. 1a), are injected into the quartz glass hydrocyclone from the tangential inlet. The micro-sphere has a relative density of 1.15. Since the diameter ratio of core to the shell reaches 42.6%, it is easily distinguished by the high-speed cameras when they are translating rapidly in the hydrocyclone. Because it’s strong anisotropic turbulence in the hydrocyclone, the turbulence scales in different areas are quite different. Therefore, the average Kolmogorov length scale, $\bar{\eta }={({\nu }^{3}/\bar{\varepsilon })}^{1/4}$, that depending on the average turbulent dissipation rate, $\bar{\varepsilon }$, at the cross section between the cylinder and the cone is taken to describe the turbulence scale and estimated to be 165 μm to 208 μm at the experimental conditions (see Supplementary Note 1). The test microsphere size (470 μm) is larger than the average Kolmogorov length scale.

The whole hydrocyclone is installed in a water-filled square Perspex jacket to minimize the optical refraction caused by the curved surface. Because the inlet position has a strong effect on the motion of particles in the hydrocyclone^37^, the micro-spheres are injected into the center of the inlet by a long syringe needle with an inner diameter of 1 mm. As the spheres pass through the needle, the quantity of the particles in the detection zone is strictly limited such that the effect of particle concentration on fluid viscosity and particle collisions can be ignored.

The hydrocyclone is set vertically and a high-speed camera fitted with a 60 mm Nikkor micro-lens is used to measure the sphere rotation (Fig. 1b). Because of the effect of centrifugal force, the spheres travel mostly in the outer spiral and boundary layer. This being the case, the white LED lights of 100 W illuminates a detection zone of approximately 25 mm × 40 mm × 10 mm located near the side wall. The frame rate is set to 10,000 Hz at a resolution of 1,024 × 1,024 pixels. Due to the tangential velocity gradient, the spheres mainly rotate around the vertical direction (named *z* coordinate). As shown in Fig. 1c, the process of overlap and separation of two black cores indicates that the micro-sphere is rotating. The sphere’s rotation speed is calculated by

1 $${\omega }_{z}=\frac{n\pi {f}_{1}}{2({N}_{f}-1)}$$

where *nπ*/2 is the rotation angle, *n* = 1, 2, 3 …, *f*_1_ is the frame rate, and *N*_*f*_ is the number of frames recording the sphere rotation.

### Effect of hydrocyclone structure on rotation speed

**Figure 2:**
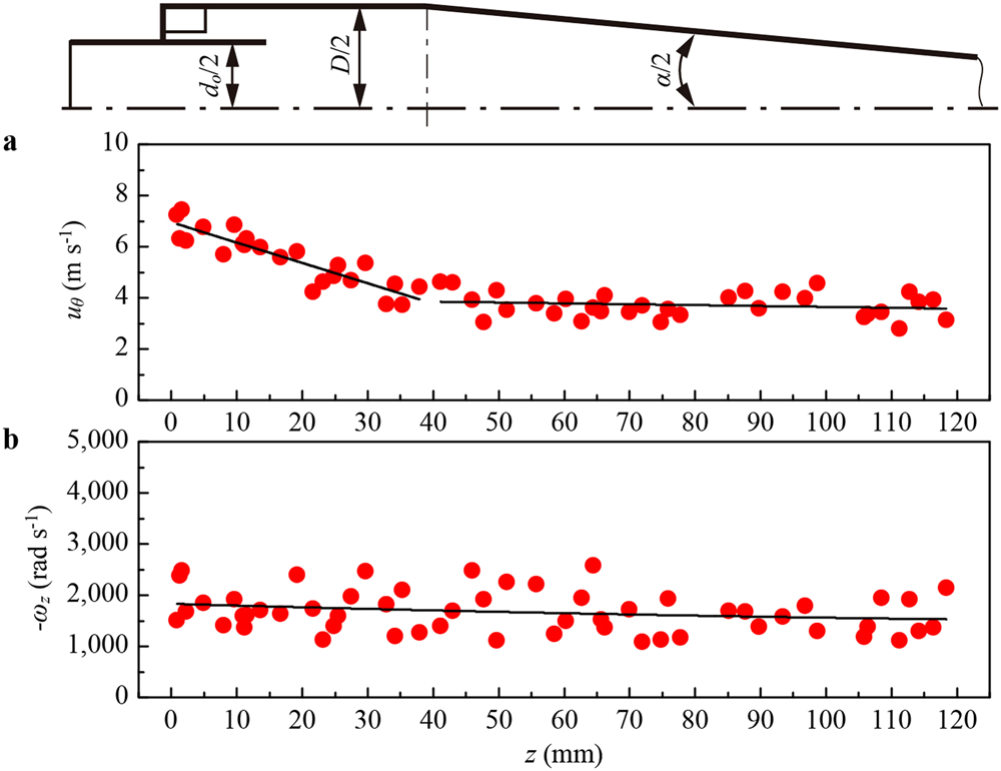
Motions of micro-spheres along the axial direction of the hydrocyclone at Re_*D*_ = 6.9 × 10^3^. (**a**) The tangential velocities *u*_*θ*_ of rotating micro-spheres. (**b**) The rotation speeds *ω*_*z*_ (z direction) of micro-spheres.

The hydrocyclone has a slender structure that consists of a cylinder and a cone. The micro-spheres are injected tangentially into the swirling field and translate following the outer spiral before finally exiting from the underflow orifice. This study only investigates the rotation of spheres caused by the tangential velocity gradient. The fluctuating term and other rotation components are negligible for the reasons that: (1) the test micro-spheres are inertial particles; (2) the turbulence intensity is about 5% (see Supplementary Table 3) that the effect of fluid velocity fluctuating on particle rotation is very small; (3) the tangential velocity gradient is much larger than the other components, and the rotation speed of sphere is proportional to the tangential velocity gradient. The tangential velocities *u*_*θ*_ of micro-spheres along the axial direction of the hydrocyclone at Re_*D*_ = 6.9 × 10^3^ are shown in Fig. 2a. The characteristic Reynolds number is defined as Re_*D*_ = *DU*_*D*_/*ν*, where *D* is the diameter of cylinder, *U*_*D*_ is the characteristic velocity and *ν* is the kinematic viscosity of fluid. Due to the factors of energy translation and dissipation, the fluid tangential velocity in the cylindrical section decreases with the distance from the inlet. However, as the inner diameter of the cone section decreases, the tangential velocity of spheres is unchanged. Therefore, the role of the conical structure is to prevent the tangential velocity decay along the axial direction of hydrocyclone, so as to keep the tangential velocity gradient. In addition, since the rotation speed of spheres is proportional to the tangential velocity gradient, the conical structure helps the spheres maintain a high rotation speed. The rotation speeds *ω*_*z*_ of micro-spheres are shown in Fig. 2b. The rotation speeds in the cylindrical section and conical section have the same distribution that is between 1,000 rad s^−1^ to 2,500 rad s^−1^ along the *z* direction, which is much larger than that in the isotropic turbulent flow.

### Effect of operating conditions on rotation speed

**Figure 3:**
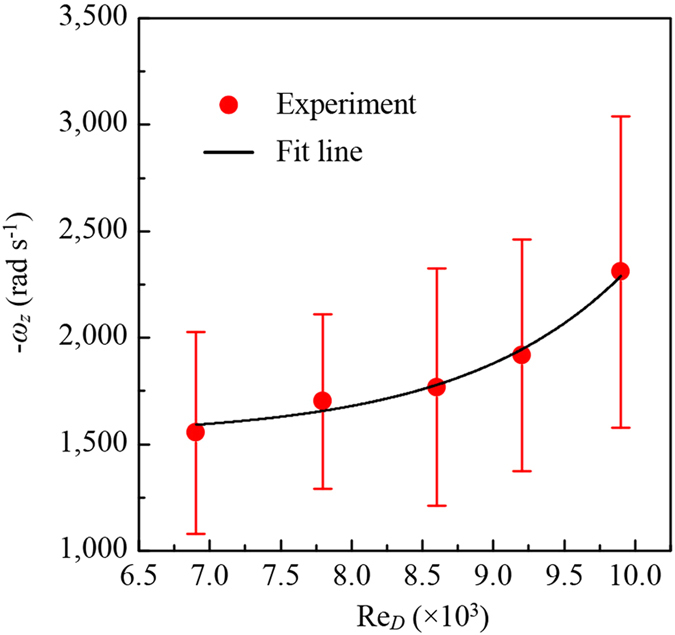
Effect of operating condition on micro-sphere rotation. The bar represents the standard deviation.

Since the rotation speeds of micro-spheres are evenly distributed in the hydrocyclone, as shown in Fig. 2b and Supplementary Figure 4, the effect of the characteristic Reynolds number Re_*D*_ on the mean rotation speed is considered and shown in Fig. 3 by plotting the mean rotation speed as a function of Re_*D*_. The standard deviation ranges from 409 rad s^−1^ to 732 rad s^−1^. The mean rotation speed increases exponentially corresponding to Re_*D*_, and the fitting equation is given by

2 $${\omega }_{z}=-0.132\exp (0.874R{e}_{D})-1536.893$$

### Numerical prediction of droplet rotation

The 3D swirling velocity field in the hydrocyclone is simulated with RSM by ANSYS Fluent 14.5. The continuous phase has a density of *ρ*_*c*_ = 580 kg m^−3^ and a dynamic viscosity of *μ*_*c*_ = 0.0002 Pa s^−1^. A discrete phase model (DPM) that considers drag force, gravity force, buoyancy force, Saffman lift force, virtual mass force and pressure gradient force is applied to predict the spiral trajectories of water droplets of sizes 10 μm, 30 μm, and 50 μm. The inlet flux of continuous phase is 0.5 m^3^ h^−1^.

By assuming the balance between the inertial and viscous forces^21^, the time scales for mico-droplets rotation are ${t}_{\omega }={\rho }_{d}{r}_{d}^{2}/(15\,{\mu }_{c})$ = 0.008 ms, 0.075 ms and 0.21 ms, where *ρ*_*d*_ and *r*_*d*_ are the density and radius of water droplets respectively. In fact, *t*_*ω*_ gives the time necessary to a particle to adjust its rotational speed to that of the surrounding fluid. According to the simulation results, the residence times are 289 ms, 201 ms and 189 ms respectively for the droplet diameter of 10 μm, 30 μm, and 50 μm. All the residence times are much larger than the relaxation time scale of microsphere rotation. Accordingly, we consider that the micro-droplets have enough time to reach the high rotation speed as the surrounding fluid in the hydrocyclone. Thus, the radial, tangential, and axial components of the sphere rotation speed can be expressed by the half of the vorticity along the spiral trajectory in the cylindrical coordinate system (***r***, ***θ***, ***z***), which is calculated by

3 $${{\boldsymbol{\omega }}}_{p}={\omega }_{r}{\boldsymbol{r}}+{\omega }_{\theta }{\boldsymbol{\theta }}+{\omega }_{z}{\boldsymbol{z}}=\frac{1}{2}(\frac{1}{r}\frac{\partial {u}_{z}}{\partial \theta }-\frac{\partial {u}_{\theta }}{\partial z}){\boldsymbol{r}}+\frac{1}{2}(\frac{\partial {u}_{r}}{\partial z}-\frac{\partial {u}_{z}}{\partial r}){\boldsymbol{\theta }}+\frac{1}{2r}(\frac{\partial (r{u}_{\theta })}{\partial r}-\frac{\partial {u}_{r}}{\partial \theta }){\boldsymbol{z}}$$

**Figure 4:**
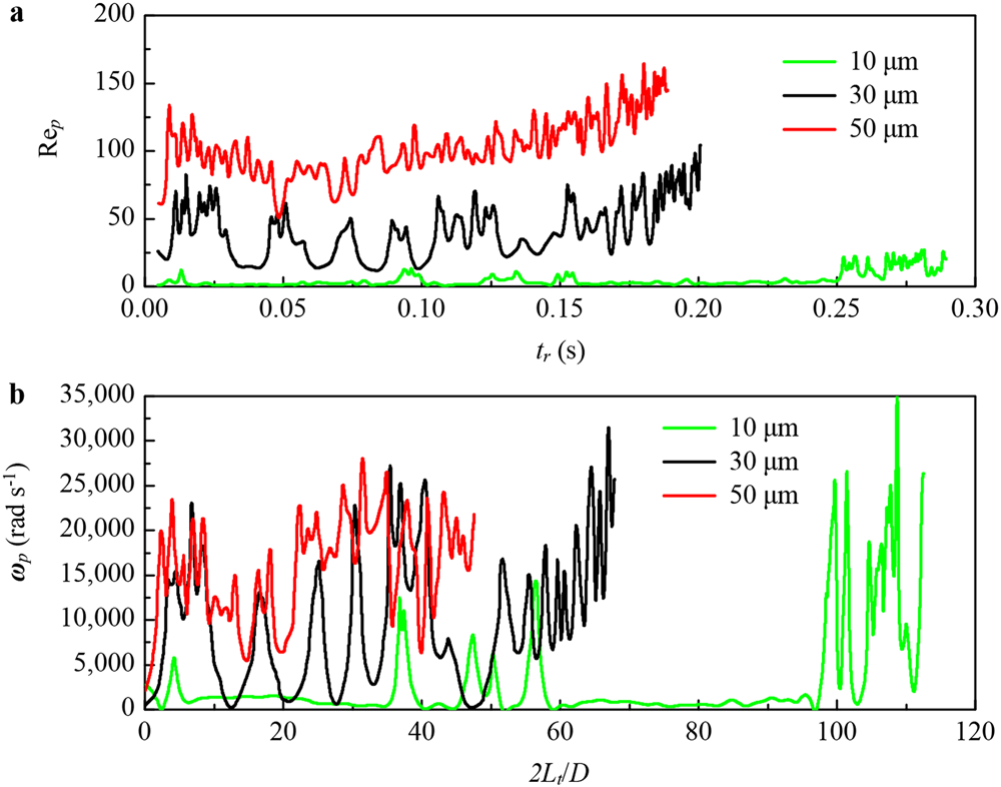
Simulation of micro-droplets in a hydrocyclone. (**a**) Particle Reynolds number of micro-droplets as they come to the underflow orifice, $R{e}_{p}={d}_{p}{V}_{p}^{\text{'}}{\rho }_{c}/\mu$. ${d}_{p}$, ${V}_{p}^{\text{'}}$, ${\rho }_{c}$, and *μ* are the droplet diameter, the relative velocity of droplets and fluid, the density of fluid and the kinetic viscosity, respectively. The resistance time *t*_*r*_ indicates the extraction time. (**b**) The rotation speed ${{\boldsymbol{\omega }}}_{p}$ of micro-droplets along the trajectories.

The period of droplet formation is confirmed to play an important role on the mass transfer of liquid-liquid extraction, which accounts for at least 30% of the total mass transfer quantities according to a theoretical analysis^38^. However, at the stage of free migration, the micro-droplets have perfect following performance, which results in a low particle Reynolds number Re_*p*_ and is not beneficial to mass transfer. The particle Reynolds number of the industrial liquid-liquid extractors is usually between 10 and 250^39^. The results of this simulation, shown in Fig. 4a, are concordant with this conclusion. Furthermore, the smaller the droplets size, the smaller the particle Reynolds number. Though the smaller droplet has good following performance, its residence time *t*_*r*_ is longer than that of the larger ones. This is because the smaller size droplets are more easily captured by the circulation flow in the hydrocyclone. As the droplets near the underflow orifice, the Re_*p*_ increases with the gradually decreasing inner diameter of the cone. From the distribution of Re_*p*_, it can be observed that the flow field in the hydrocyclone has the same condition as a conventional extractor for mass transfer.

Different from the isotropic turbulent flow in most extraction columns, the fluid in a hydrocyclone is a 3D anisotropic swirling shear flow, which causes the particles immersed in it to rotate. The rotation speeds of micro-droplets, based on equation (3), along their spiral trajectories are shown in Fig. 4b. Due to the strong shear forces, the rotation speeds of micro-droplets in the hydrocyclone could be more than 10,000 s^−1^, which is much larger than those in isotropic turbulent flows^25, 27, 29^. The high-speed rotation leads to a strong interface turbulence and inner circulation flow within micro-droplets, which makes the two sides of the interface maintain a high mass transfer driving force. In addition, the trajectories of micro-droplets have lengths *L*_*t*_ of more than 1,000 mm, which is exceed 50 times of the inner radius D/2 and indicates that the mixing length of the continuous phase. Therefore, the probability of extractant micro-droplets capturing solute molecules and ions is much higher. As a result, the extraction efficiency is improved.

### Industrial application results

**Figure 5:**
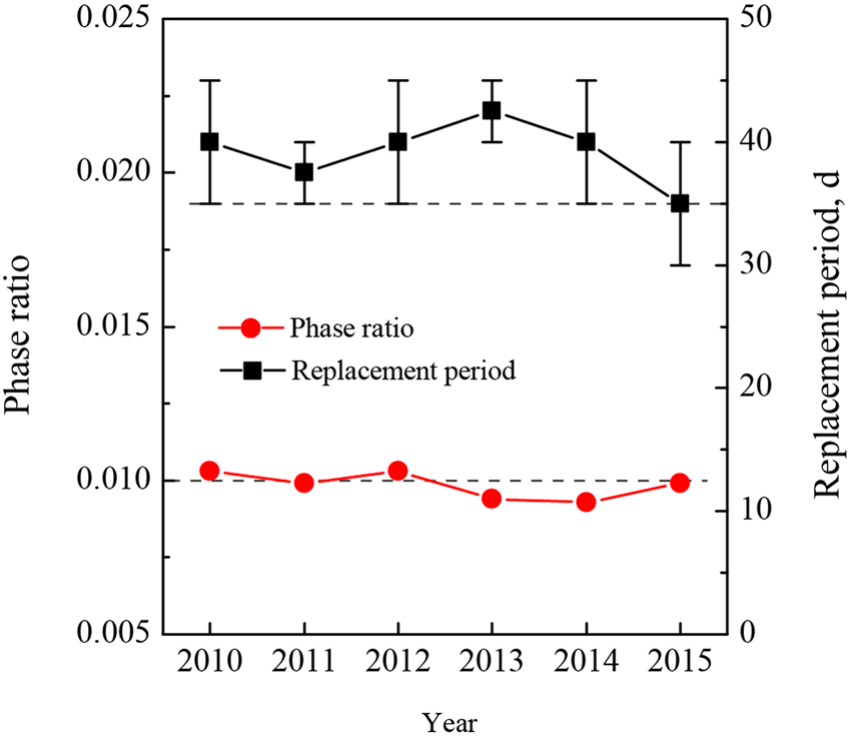
Operation of the hydrocyclone in industry. The phase ratio is calculated by the annual consumption of water divided by that of the C_4_ feed. The catalyst replacement period of the reactor protection filter is plotted by the average time interval in a year, and the error bar illustrates the shortest and longest replacement periods.

The operation of the hydrocyclone of the MTBE unit of the PetroChina Karamay Petrochemical Company for the past six years was investigated. The average C_4_ feed throughput of the unit is maintained at 7.7–9.6 t h^−1^, and the water used to extract the impurities is in the range of 0.079–0.090 kg h^−1^. As shown in Fig. 5, the phase ratio is approximately 0.01, which is similar with the previous reports^7^. The general maintenance cycle of the unit is yearly, and the catalysts of the reactors will be replaced each time. Two reactor protection filters utilize the same catalyst with the reactors operated alternately to consume the impurities. Their catalyst replacement period is also kept stable with an average of 40 d as formerly reported (Fig. 5), which makes the reactor’s catalyst lifetime reach the maintenance cycle.

## Discussion

Determined by the principle of identifying the sphere rotation, the minimum detection angle is π/2. As a result, the error of rotation speed is mainly caused by the limited frame rate of high speed camera that the recording rotation angle nπ/2 may deviate to the real value. The error angle, *α*_*E*_, of recording rotation angle could be calculated by the equation:

4 $${\alpha }_{E}=\frac{{\omega }_{z}}{f}$$

The maximum error of rotation speed, *E*_*m*_, is calculated by the equation:

5 $${E}_{m}=\frac{4{\alpha }_{E}}{n\pi }=\frac{4{\omega }_{z}}{n\pi f}$$

Since the rotation speed is *ω*_*z*_  ≈  1000–2500 rad/s, the frame rate is *f* = 10,000 Hz and usually n ≥ 2, the maximum error of rotation speeds is *E*_*m*_ = 6.4%–15.9%.

**Figure 6:**
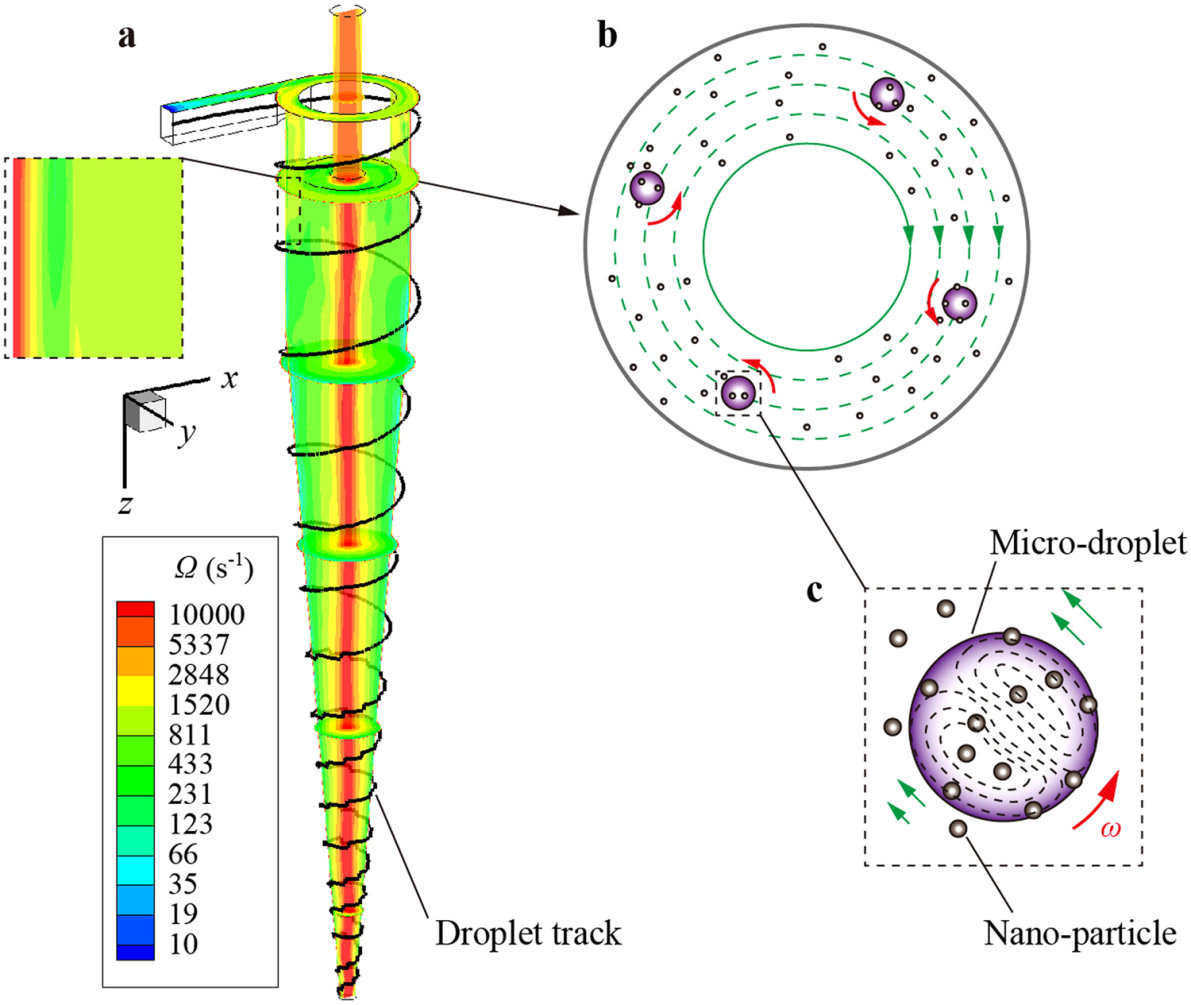
Process of micro-droplet extracting nano-particles in a hydrocyclone. (**a**) The numerical results of vorticity distribution in the hydrocyclone. The micro-droplet travels along a spiral trajectory. (**b**) Schematic of the rotating micro-droplets capturing the solute molecules or ions. (**c**) Schematic of the inner circulation flow in a rotation droplet.

Based on the characteristics of the shear flow in a hydrocyclone and the rotation behavior of micro-droplets along spiral trajectories, we conclude that the mechanism of extraction intensification by hydrocyclone is as follows: (1) The swirling flow in a hydrocyclone has a strong eddy diffusion effect that intensifies the convectional mass transfer of the solute in the continuous phase (Fig. 6a). (2) The extractant micro-droplets travel along spiral trajectories, which make the continuous phase and the dispersed phase have a long mixing length, which results in an increased probability of the dispersed phase capturing solute particles. (3) The strong shear flow makes the micro-droplets rotate rapidly (Fig. 6b), which causes strong interface turbulence and inner circulation flow in the droplets (Fig. 6c). Therefore, the droplets have low mass transfer resistance until they reach saturation.

## Methods

### Experimental

The highly monodisperse micro-spheres are fabricated in a two-stage glass capillary microfluidic device^40, 41^ (Supplementary Figure 1a–d). The components of each phase are given in Supplementary Table 1. The core’s black color is due to the carbon black ink. The oil shell of emulsion droplet with photoinitiator is solidified in 3 minutes when exposed to UV light. The rotation behavior of the micro-spheres is distinguished by the overlap and separation of cores (Supplementary Figure 1e). The experiments of measuring rotation of the micro-spheres are conducted in the conventional circulation separation system of a hydrocyclone. The apparatus is shown in Supplementary Figure 2. The testing particles in the feeder are carried by the flow into the inlet center of the hydrocyclone through a long syringe needle with an inner diameter of 1 mm. The hydrocyclone is made of optical glass with a cylindrical diameter of 25 mm and a conical angle of 10°. The other structural parameters are shown in Supplementary Table 2. To investigate the effect of inlet flow rate on particle rotation speed, five operating conditions are tested (Supplementary Table 3). Because of the stochastic orientation of the two cores, most of the micro-spheres do not provide their rotation information. The rotating spheres suitable for analysis are picked out manually, and their translation and rotation speeds are determined with the help of image analyzing software, e.g., *Image-pro plus*. A typical rotating micro-sphere is shown in the Supplementary Video.

### Numerical

The simulation is conducted in the commercial package *ANSYS Fluent 14*.*5*. The 3D velocity field and droplet trajectories are calculated by RSM and DPM, respectively. Non-equilibrium wall functions are used for the near-wall treatment. The Semi-Implicit Method for Pressure Linked Equation Consistent (SIMPLEC) algorithm is used to solve the pressure-velocity coupling equation. Pressure Staggered Option (PRESTO!) is employed for pressure discretization. The discretization of other conservation equations is solved by the Quadratic Upwind Interpolation of Convective Kinematics (QUICK) scheme. Outflow boundary condition type is specified at the overflow and underflow orifices with a split ratio (the ratio of underflow rate to inlet flow rate) of 0.1. The drag coefficient, ${C}_{D}={a}_{1}+{a}_{2}/Re+{a}_{3}/R{e}^{2}$, for smooth particles given by Morsi and Alexander^40^ is taken to correct the drag force, where ${a}_{1}$, ${a}_{2}$ and ${a}_{3}$ are constants that apply over several ranges of fluid Reynolds number, Re. The computational domain has 603,540 hexahedral cells, which includes the refined boundary layer (Supplementary Figure 5).

## Electronic supplementary material

Dataset 1

Rotation of micro-sphere
