# Supplementary material for: Liquid-liquid extraction intensification by micro-droplet rotation in a hydrocyclone
Source: Sci Rep. 2017 Jun 2;7:2678. doi: 10.1038/s41598-017-02732-x (PMC5457425; doi:10.1038/s41598-017-02732-x)
Supplement: Supplementary file 1 — Dataset 1 [file 41598_2017_2732_MOESM1_ESM.doc]

# **Liquid-liquid extraction intensification by micro-droplet rotation in a hydrocyclone (supplementary information)**

*Yuan Huang*^1^*, Hua-lin Wang*^1*^*, Yu-quan Chen*^2^*, Yan-hong Zhang*^1^*, Qiang Yang*^1^*, Zhi-shan Bai*^1^*, Liang Ma*^1^

^1^State-Key Laboratory of Chemical Engineering, East China University of Science and Technology, Shanghai 200237, PR China

^2^PetroChina Karamay Petrochemical Company, Kelamayi 834003, PR China

# **Supplementary figures**


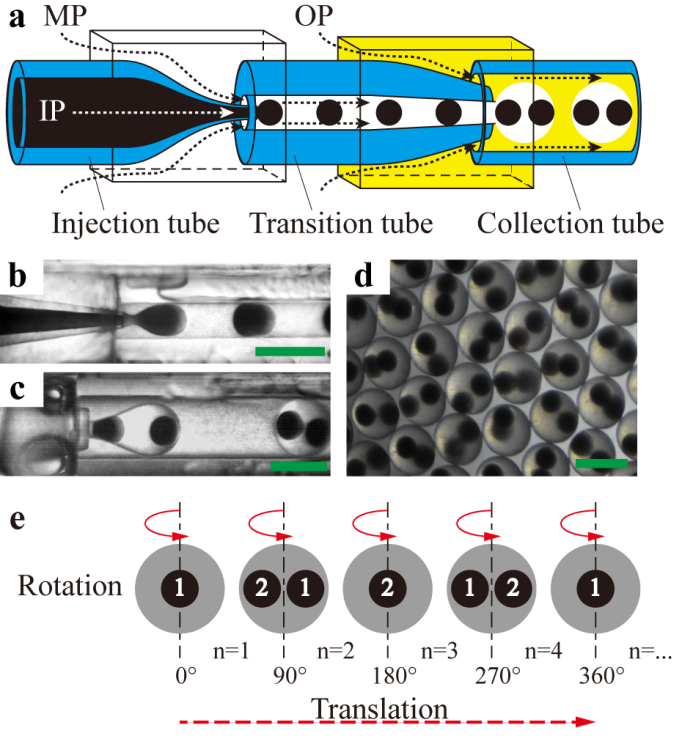


**Supplementary Figure 1: Process of fabricating testing micro-spheres.** **(a)** Schematic diagram of microfluidic device used to generate monodisperse W/O/W emulsions with double black cores. **(b-c)** Optical micrographs of showing the generation of black cores (b) and oil shell (c). **(d)** The solidified micro-spheres. **(e)** The principle of distinguishing micro-sphere rotation.


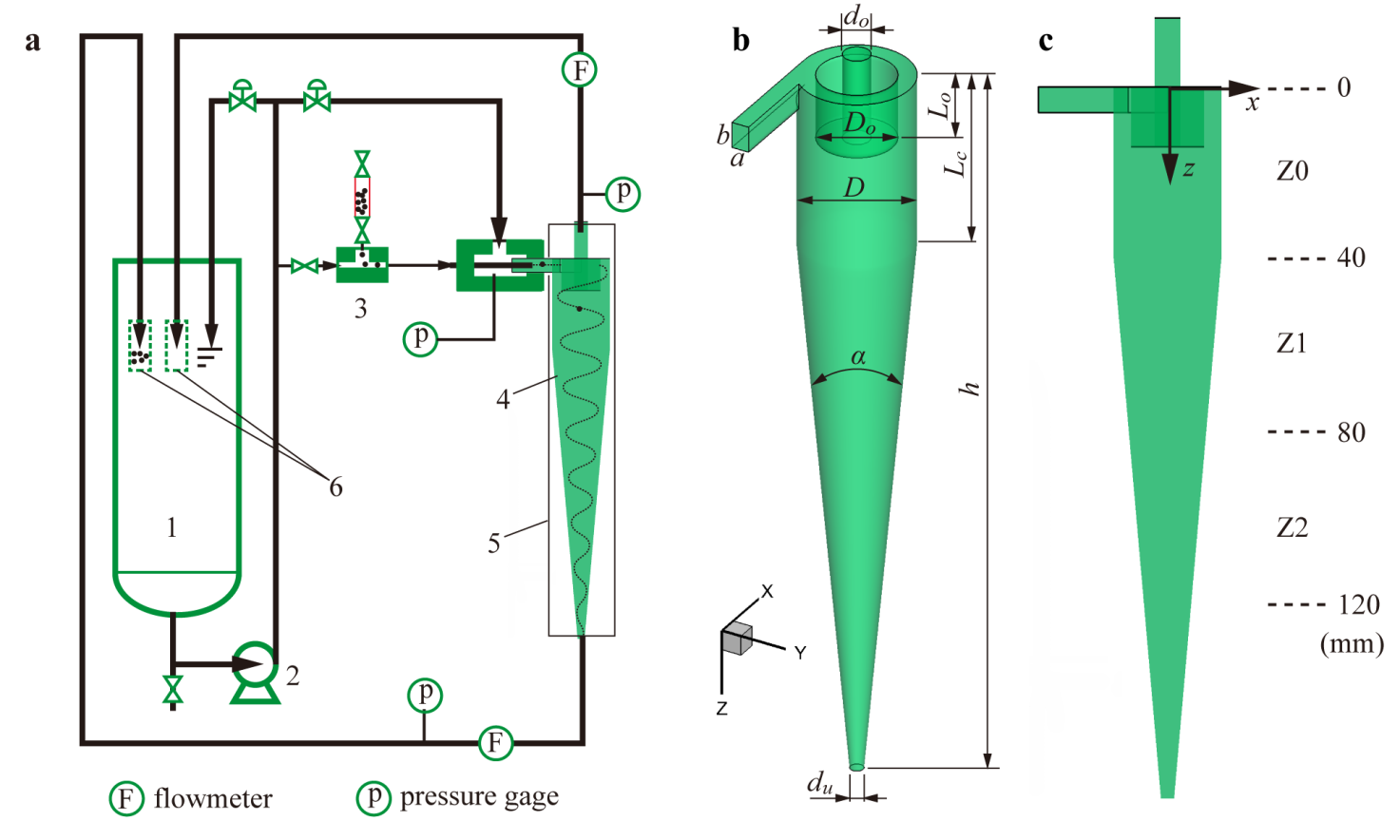


**Supplementary Figure 2: Experimental apparatus.** **(a)** The circulation separation system of hydrocyclone. 1-tank; 2-turbulence pump; 3-testing particle feeder; 4-hydrocyclone; 5-square Perspex water jacket; 6-filter screens. The water in the tank is pumped into the quartz glass hydrocyclone, and then return to tank through the overflow and underflow pipes respectively. A water-filled square Perspex jacket surrounds the whole hydrocyclone to reduce optical distortion. The testing micro-spheres in the feeder are carried by the flow into the inlet center of the hydrocyclone through a long syringe needle of 1mm in inner diameter, and then discharged from the underflow orifice. The needle makes the testing particles enter the flow field in turn, which strictly limits the quantity of particles in the detection zone, and eliminates the effect of particle concentration on fluid viscosity and particle collision. The discharged particles are collected with filter screens at the end of the underflow and overflow pipes. The flow rate is regulated through the valves. **(b)** Structural Schematic of the hydrocyclone. Its parameters are showed in supplementary table 2. The origin of Cartesian coordinates is fixed at the top center of the hydrocyclone. **(c)** Detection zone. Limited by the view field of cameras, the detection zone is divided into three sections (40 mm long for each section) along the axis direction (z), which are marked Z0 to Z2 continuously. Because the inner diameter of cone section near the underflow orifice is too small that results in larger measurement errors, the section of z >120 mm doesn’t considered.


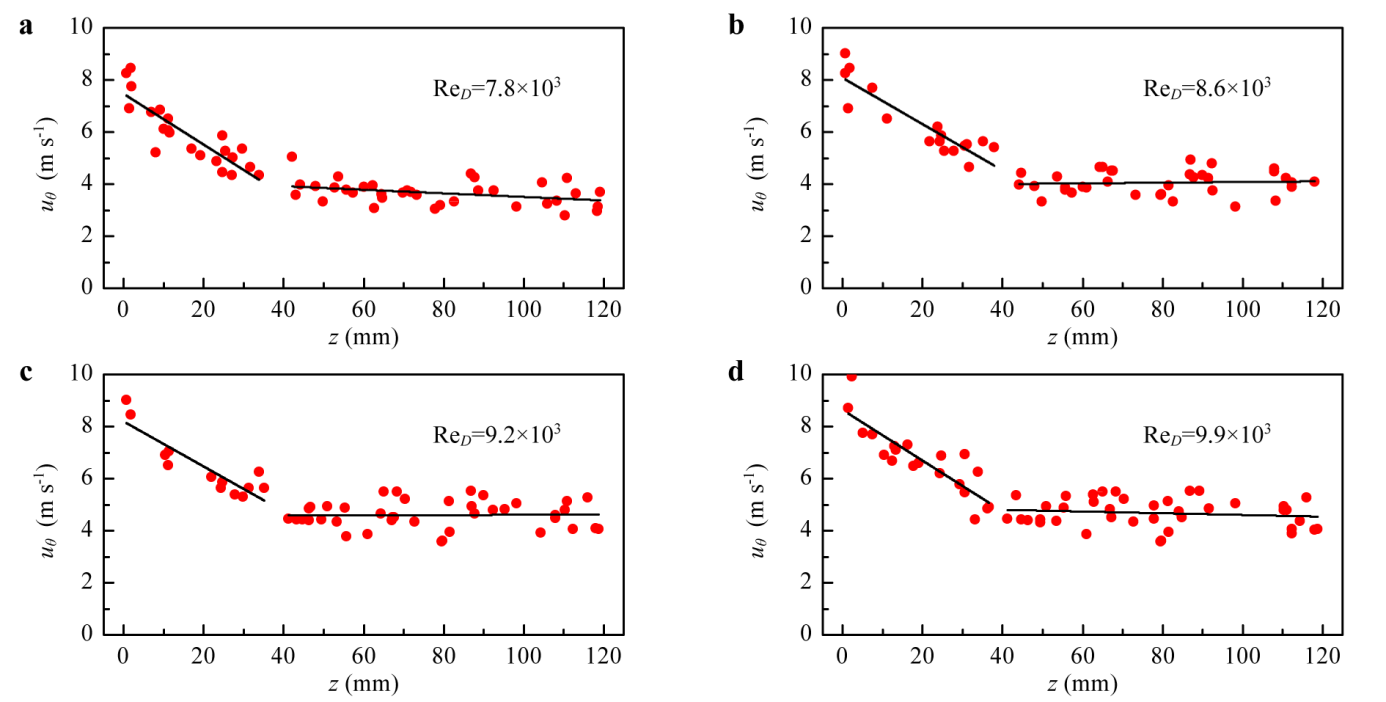


**Supplementary Figure 3: The tangential velocity distribution at various operating conditions. (a-d)** Due to the specific structures, the rotation speed of micro-sphere decreases in the cylindrical sectiong but keep stable in the conical section as it comes to the underflow orifice.


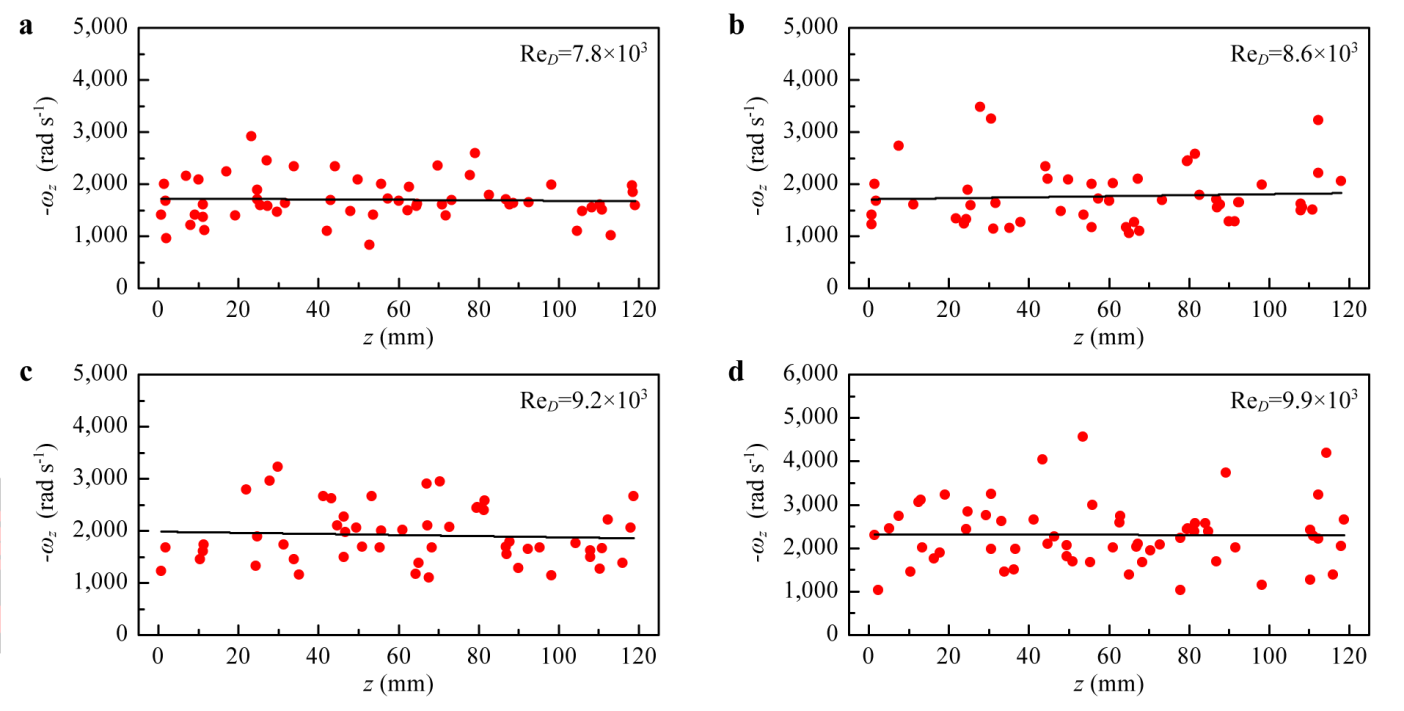


**Supplementary Figure 4: The rotation speed distribution of at different operating conditions. (a-d)** Because the rotation speed of micro-spheres is related to tangential velocity gradient, all the conditions show that the rotation speed remains stable along the axial direction.


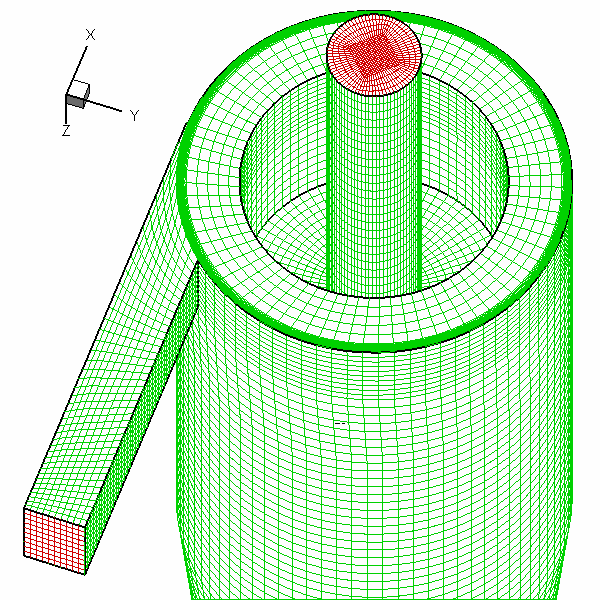


**Supplementary Figure 5: Structural mesh of hydrocyclone.** The hexahedral meshes of computational domain are generated in *Gambit*. Considering the effect of boundary layer, the mesh near the side wall is refined with a strategy that the first layer is set to 0.005 mm with an increasing factor 1.3 and 8 layers of total. The origin is set to the center of the top cover.


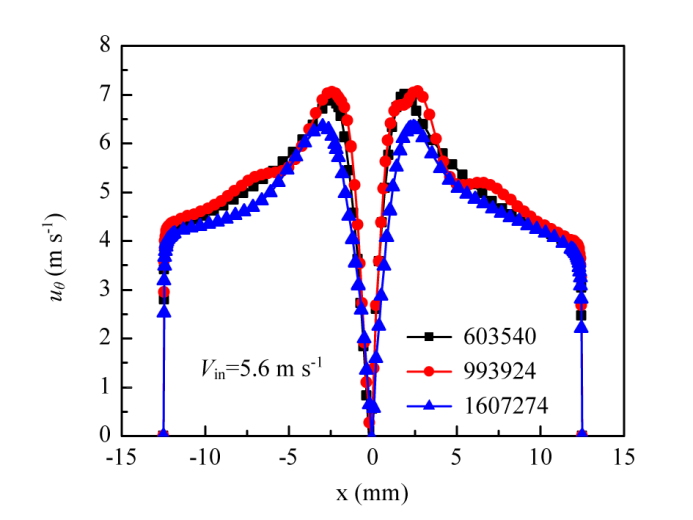


**Supplementary Figure 6: Tangential velocity distribution of cylinder part of hydrocyclone under different mesh densities.** In order to verify the mesh independence, the tangential velocities of three mesh densities are compared. The mesh densities are N_1_=603540, N_2_=993924and N_3_=1607274. The tangential velocities showed in the figure are at the height of z=40 mm. Base on the result of mesh density N_1_, the mean errors are 3.9 % and 6.8% respectively. Thereby, the mesh density of N_1_=603540 is used in this study.

# **Supplementary tables**

**Supplementary Table 1: Components of emulsion droplets.**

| Item | Components and content |
| --- | --- |
| Inner flow(IF) | Water+ Pluronic F127 (1%w/v)+ glycerol (5%w/v) + carbon black ink (3%v/v) |
| Middle flow(MF) | 1,6-Bis(acryloyloxy)hexane+ polyglycerol polyricinoleate ( 5%v/v)  +2-hydroxy-2-methyl-1-phenyl-1-propanone (1%v/v) |
| Outer flow (OF) | Water+ Pluronic F127 (1%w/v)+ glycerol (5%w/v) |

**Supplementary Table 2: Structural parameters of hydrocyclone.**

| *D*，mm | *b*/*a* | *d*_o_/*D* | *L_o_*/*D* | *L_c_*/*D* | *α*/(°) | *d*_u_/*D* |
| --- | --- | --- | --- | --- | --- | --- |
| 25 | 1.5 | 0.24 | 0.57 | 1.6 | 10 | 0.12 |

**Supplementary Table 3: Operating conditions of hydrocyclone.**

|  | I | II | III | IV | V |
| --- | --- | --- | --- | --- | --- |
| Press drop ∆*P*, MPa | 0.10 | 0.14 | 0.18 | 0.22 | 0.26 |
| Inlet flow rate *Q_i_*, L min^-1^ | 8.1 | 9.1 | 10.1 | 10.8 | 11.6 |
| Mean inlet velocity *U_i_*, m s^-1^ | 5.6 | 6.3 | 7.0 | 7.5 | 8.1 |
| Reynolds number Re*_D_*,×10^3^ | 6.9 | 7.8 | 8.6 | 9.2 | 9.9 |
| Split ratio *R_u_*, % | 21.5 | 20.8 | 20.2 | 19.7 | 18.8 |
| Average turbulence intensity , % | 5.30 | 5.23 | 5.16 | 5.12 | 5.07 |
| Average Kolmogorov length scale , μm | 208 | 193 | 180 | 172 | 165 |

Where ∆*P* is the between the inlet and the overflow, *R_u_* is flow rate ratio between underflow and the inlet.

# **Supplementary video 1**

A typical rotation of test micro-sphere at the operation condition IV. The inner double black cores overlap and separate alternatively which indicates the rotation of the micro-sphere. The rotation speed *ω_z_* is calculated to be 2617 rad s^-1^.

# **Supplementary note 1. *Estimation of the average Kolmogorov length scale in hydrocyclone.***

The characteristic velocity of fluid at the cross section between the cylinder and the cone is calculated by the equation:

$$U_{D}=\frac{Q_{i}}{\pi R^{2}}$$

where *Q_i_* is the inlet flux, m^3^/s; *R* is the radius of the cylinder section.

Then, the characteristic Reynolds number is calculated by the equation:

$$\mathrm{Re}_{D}=\frac{DU_{D}}{\nu}$$

where *ρ* is the density of fluid; *D* is the diameter of cylinder or cross section; *ν* is the kinematic viscosity.

The turbulence intensity at the core of a fully-developed ductile flow can be estimated from the following formula from an empirical correlation for pipe flows:

$$I=0.16(\mathrm{Re}_{D})^{-1/8}$$

The turbulence length scale, *l*, is a physical quantity related to the size of the large eddies that contain the energy in turbulent flows. In fully-developed duct flows, *l* is restricted by the size of the duct, since the turbulent eddies cannot be larger than the duct. An approximate relationship between *l* and the physical size of the duct is

$$l=0.07D$$

The relationship between the turbulent kinetic energy, *k*, and turbulence intensity, *I*, is

$$k=\frac{3}{2}\left(U_{D}I\right)^{2}$$

The turbulent dissipation rate is calculated by the formula:

$$\varepsilon=C_{\mu}^{3/4}\frac{k^{3/2}}{l}$$

where *C_μ_* is an empirical constant specified in the turbulence model (approximately 0.09). The formulas (3) to (6) is referred to the help document of ANSYS fluent.

The average Kolmogorov length scale is calculated by the formula:

$$\bar{\eta}=\left(\frac{\nu^{3}}{\varepsilon}\right)^{1/4}$$
