# Supplementary figures and images for: Liquid-liquid extraction intensification by micro-droplet rotation in a hydrocyclone
Source: Sci Rep. 2017 Jun 2;7:2678. doi: 10.1038/s41598-017-02732-x (PMC5457425; doi:10.1038/s41598-017-02732-x)

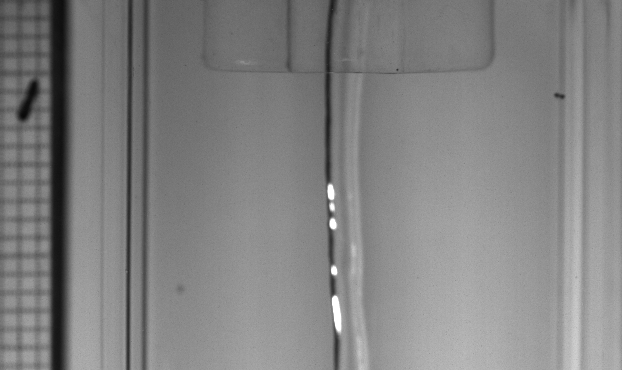

Supplement: Supplementary file 2 — Rotation of micro-sphere [file 41598_2017_2732_MOESM2_ESM.gif]
